# Incidental irradiation of the regional lymph nodes during deep inspiration breath-hold radiation therapy in left-sided breast cancer patients: a dosimetric analysis

**DOI:** 10.1186/s12885-022-09784-x

**Published:** 2022-06-21

**Authors:** Jule Wolf, Steffen Kurz, Thomas Rothe, Marco Serpa, Jutta Scholber, Thalia Erbes, Eleni Gkika, Dimos Baltas, Vivek Verma, David Krug, Ingolf Juhasz-Böss, Anca-Ligia Grosu, Nils H. Nicolay, Tanja Sprave

**Affiliations:** 1 Department of Radiation Oncology, University Hospital of Freiburg, Robert-Koch-Strasse 3, 79106 Freiburg, Germany; 2 German Cancer Research Center (Dkfz), German Cancer Consortium (DKTK) Partner Site Freiburg, Im Neuenheimer Feld 280, 69120 Heidelberg, Germany; 3 Department of Obstetrics and Gynecology, Medical Center, University of Freiburg, Freiburg, Germany; 4 Department of Radiation Oncology, MD Anderson Cancer Center, Houston, TX USA; 5 Department of Radiation Oncology, University Hospital Schleswig-Holstein, Arnold-Heller-Str. 3, 24105 Kiel, Germany; 6 Department of Molecular and Radiation Oncology, German Cancer Research Center (Dkfz), Im Neuenheimer Feld 280, 69120 Heidelberg, Germany

**Keywords:** Breast cancer, Deep inspiration breath-hold radiation therapy, Incidental irradiation, Lymph nodes

## Abstract

**Background:**

Radiotherapy using the deep inspiration breath-hold (DIBH) technique compared with free breathing (FB) can achieve substantial reduction of heart and lung doses in left-sided breast cancer cases. The anatomical organ movement in deep inspiration also cause unintended exposure of locoregional lymph nodes to the irradiation field.

**Methods:**

From 2017–2020, 148 patients with left-sided breast cancer underwent breast conserving surgery (BCS) or mastectomy (ME) with axillary lymph node staging, followed by adjuvant irradiation in DIBH technique. Neoadjuvant or adjuvant systemic therapy was administered depending on hormone receptor and HER2-status.

CT scans in FB and DIBH position with individual coaching and determination of the breathing amplitude during the radiation planning CT were performed for all patients. Intrafractional 3D position monitoring of the patient surface in deep inspiration and gating was performed using Sentinel and Catalyst HD 3D surface scanning systems (C-RAD, Catalyst, C-RAD AB, Uppsala, Sweden). Three-dimensional treatment planning was performed using standard tangential treatment portals (6 or 18 MV). The delineation of ipsilateral locoregional lymph nodes was done on the FB and the DIBH CT-scan according to the RTOG recommendations.

**Results:**

The mean doses (D_mean_) in axillary lymph node (AL) level I, II and III in DIBH were 32.28 Gy (range 2.87–51.7), 20.1 Gy (range 0.44–53.84) and 3.84 Gy (range 0.25–39.23) vs. 34.93 Gy (range 10.52–50.40), 16.40 Gy (range 0.38–52.40) and 3.06 Gy (range 0.21–40.48) in FB (*p* < 0.0001). Accordingly, in DIBH the D_mean_ for AL level I were reduced by 7.59%, whereas for AL level II and III increased by 22.56% and 25.49%, respectively.

The D_mean_ for the supraclavicular lymph nodes (SC) in DIBH was 0.82 Gy (range 0.23–4.11), as compared to 0.84 Gy (range 0.22–10.80) with FB (*p* = 0.002). This results in a mean dose reduction of 2.38% in DIBH.

The D_mean_ for internal mammary lymph nodes (IM) was 12.77 Gy (range 1.45–39.09) in DIBH vs. 11.17 Gy (range 1.34–44.24) in FB (*p* = 0.005). This yields a mean dose increase of 14.32% in DIBH.

**Conclusions:**

The DIBH technique may result in changes in the incidental dose exposure of regional lymph node areas.

## Introduction

Incidental irradiation of cardiac structures in left-sided breast cancer increases the risk of subsequent ischaemic cardiac events [1]. Notably, any Gy increase in mean cardiac dose correlates linearly with a 7.4% increase in non-threshold cardiac events [2]. Different absorbed doses and irradiated volumes result in a variety of pathophysiologic events: macrovascular damage to the coronary vessels such as atherosclerosis and myocardial infarction, or microvascular damage with valvular heart disease and heart failure [3, 4]. Disturbingly, breast cancer patients who developed cardiac disease after initial cancer diagnosis have a higher risk of recurrence and cancer-specific death [5]. In particular, dose-dependent vulnerability of the left ventricle and all coronary segments justifies more rigorous dose reduction [6–9].

Currently, deep inspiration breath hold technique (DIBH) in the supine position allows reproducible cardiac shift from the irradiation field. Therefore, it is a widely used protective heart approach [10]. DIBH can be performed by tangential 3D-conformal radiotherapy (3DRT) or rotational/multiangle intensity-modulated radiotherapy (IMRT/VMAT) [11].

Tangential 3DRT of the mammary gland tissue often unintentionally includes the locoregional lymph nodes in the radiation fields with therapeutic dose. This dose may be sufficient for the eradication of microscopic tumor residues. This hypothesis is supported by the results of the ACOSOG Z0011 study, where patients with 1–2 involved sentinel lymph nodes upon sentinel lymph node dissection had similar axillary recurrence rates whether they received secondary axillary lymph node dissection or not [12]. However, a retrospective review revealed that high tangents and supraclavicular irradiation were used in a significant proportion of patients [13].

We propose that DIBH may lead to differences in the incidental radiation dose of the axillary, supraclavicular and internal mammary lymph nodes due to changes in their position [14, 15] in relation to the tangential radiation portals.

In the absence of published data in this regard from randomized trials of DIBH vs free breathing (FB) RT in the supine position, reporting institutional experiences is necessary. The goal of this single-institutional retrospective study was to investigate dosimetric differences of incidental locoregional lymph nodes irradiation between DIBH and FB for left-sided breast cancer patients.

## Materials and methods

### Patient selection and treatment planning

From December 2017 to July 2020, 148 out of 247 patients with left-sided or bilateral breast cancer which were screened for irradiation in DIBH technique were included in this analysis. The majority of patients (131 patients, 88.5%) received 3DRT in DIBH. The remaining 17 (11.5%) patients received RT in FB either due to suboptimal compliance or lack of dosimetric benefits of DIBH.

BCS or mastectomy with axillary lymph node staging was performed according to institutional protocols. Systemic therapy was administered according to current guidelines [16] and individual recommendations of the multidisciplinary tumor board. A tumor bed boost was administered for all premenopausal patients or for postmenopausal patients with additional risk factors (tumor stage ≥ T2, extensive intraductal component, grade 3, HER2-positive or triple-negative tumors).

All patients received coaching for DIBH in the CT room using a Surface Image Guided RT (SGRT) system (C-RAD, Catalyst, C-RAD AB, Uppsala, Sweden). The patients were asked to take a deep breath and hold it for a duration of 20 s. The width of the gating window was set to 5 mm. All patients received two CT scans with a slice thickness of 2 mm (Brilliance, CT Big Bore, Philips, Cleveland, OH) in FB and DIBH. Treatment planning (Oncentra MasterPlan, Nucletron, Veenendaal, The Netherlands and/or Eclipse™ planning systems (Varian Medical Systems)) was carried out using standard tangential treatment portals (6 or 18 MV; Synergy; Elekta, Crawley, United Kingdom).

Adjuvant WBI or thoracic wall RT was delivered using either moderate hypofractionation (40.05 Gy in 15 fractions) or conventional fractionation (50.00–50.40 Gy in 25–28 fractions). Boost irradiation was delivered sequentially (10–16 Gy in 5–8 fractions), with simultaneous integrated boost (58.80–61.60 Gy in 25–28 fractions) or IORT (single dose of 20 Gy with 50-kV photons [17]).

During follow up, all patients were examined every three to six months for the first two years in the radiation oncology department, followed by annual visits thereafter. Breast ultrasound was performed every 6 months for the first three years. Mammograms were obtained six months after WBI, and yearly after the first mammography. Suspected recurrences were biopsy confirmed.

### Statistical analysis

**Fig. 1:**
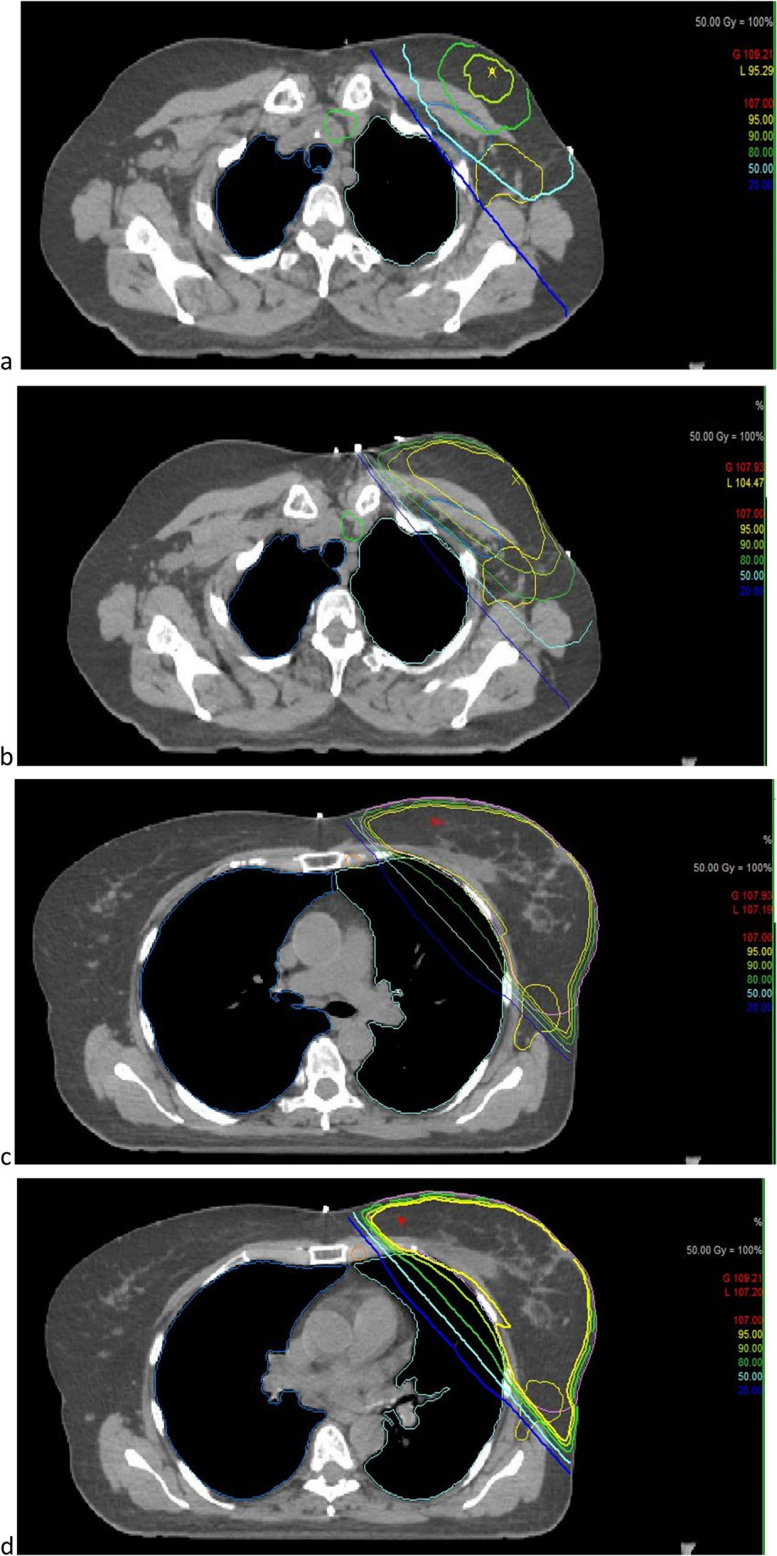
Delineation of locoregional lymph nodes and isodose distribution. **a-c**: Delineation of the supraclavicular (green), axillary lymph levels I (yellow) and II (light blue axillary lymph nodes in level III (blue), internal mammary (orange). a + c: CT scan in DIBH; b + d: CT scan in FB; a**-**d: Visualization of isodose distribution in DIBH and FB

For the planned dosimetric evaluation, lymph nodes levels of interest (axillary lymph node levels I, II, III, supraclavicular (SC) and internal mammary (IM) lymph nodes) as well as the contralateral breast were retrospectively delineated (Fig. 1). The delineation was done on both the FB and the DIBH scan according to the RTOG recommendations [18]. DVH parameters were then extracted for all delineated structures (volume, D_mean_, D50%, D_max_, D_min_, V_30_, V_40_).

Data are reported as a mean, median (range), and frequencies. For all dosimetric parameters, mean values and their corresponding ranges as well as the relative dose reduction were determined. DVH parameters of the FB vs. DIBH plans were compared using a Wilcoxon signed-rank test. *P*-values < 0.05 were considered statistically significant. Effect size was assessed according to Cohen (1988) [19]. Analysis was performed using SPSS version 27 (IBM, Armonk, NY, USA).

## Results

Altogether, 148 patients with 296 CT scans were analyzed. Baseline characteristics are shown in Table 1. Most patients had T1 (67.6%, *n* = 100) and N0 stage (87.8%, *n* = 130) with positive estrogen and progesterone receptor status. Poor differentiation (G3) and Ki-67 > 20% were present in 25.7% and 41.9 of patients, respectively. A minority of patients required re-resection to achieve clear margins (12.8%). Only four patients received mastectomy (2.7%). About 23% of patients received hypofractionated radiotherapy.

**Table 1:** Patient, tumor and treatment characteristics for patients treated using deep inspiration breath hold technique for whole breast or thoracic wall irradiation in our institution between 2017 and 2019 (*n* = 148). Staging of breast cancer was based on the 7th Edition of the UICC TNM classification

Total patients: *n* = 148	n	%
BCS	143	96.6
ME	4	2.7
no surgery	1	0.7
**TNM classification**		
PTis	17	11.5
pT1	100	67.6
pT2	29	19.6
pT3	1	0.7
pT4	1	0.7
N0	130	87.8
N1a	18	12.2
M0	147	99.3
M1	1	0.7
**Resection status**		
R0	128	86.5
initially R1, after second resection R0	19	12.8
no resection	1	0.7
**Grading**		
G1	26	17.6
G2	80	54.1
G3	38	25.7
not specified	4	2.7
**Hormone receptor status**		
**ER**		
positive	131	88.5
negative	17	11.5
not specified	0	0
**PR**		
positive	121	81.8
negative	27	18.2
not specified	0	0
**Ki-67 Score**		
low (< 10%)	13	8.8
intermediate (10–25%)	58	39.2
high (> 25%)	62	41.9
not specified	15	10.1
**HER2 status**		
0	47	31.8
1 +	52	35.1
2 +	20	13.5
3 +	13	8.8
not specified	16	10.8
**TNBC**	10	6.8
**Radiotherapy**		
Conventional fractionation	114	77
Moderate hypofractionation	34	23
SIB	46	31.1
IORT	53	35.8
3DRT	148	100
DIBH	131	88.5
FB	17	11.5
**SLND**		
Yes	122	82.4
No	18	12.2
ALND	8	5.4
**Chemotherapy**		
Neoadjuvant	24	16.2
Adjuvant	32	21.6
**Endocrine therapy**	116	78.4

*Abbreviation*: *ALND* Axillary lymph node dissection, *BCS* Breast conserving surgery, *3DRT* 3D-conformal radiotherapy, *ER* Estrogen receptor, *IORT* Intraoperative radiotherapy, *ME* Mastectomy, *PR* Progesterone receptor, *SIB* Simultaneous integrated boost, *SLND* Sentinel lymph node dissection, *TNBC* Triple negative breast cancer, *p* Pathological

### Axillary lymph node levels I, II and III

The DVH parameters for levels I-III, SC and IM are summarized in Table 2.

**Table 2:** Comparison of selected DVH parameters for locoregional lymph node levels in axillary levels I-III, supraclavicular and internal mammary region and contralateral breast in DIBH and FB technique

DVH parameter	FB		DIBH		Change [%]	*p*-value	Effect size r
	**Mean value**	**Range**	**Mean value**	**Range**			
**Axillary level I**							
Volume [ccm]	62.62	17.90–126.90	62.83	16.70–119.40	0.34	0.210	0.103
Dmean [Gy]	34.93	10.52–50.40	32.28	2.87–51.7	-7.59	< 0.0001	0.375
D50% [Gy]	39.02	2.75–52.05	35.42	1.64–53.35	-9.23	< 0.0001	0.388
Dmax [Gy]	51.4	39.71–64.12	51.27	39.10–63.95	-0.25	0.997	0
Dmin [Gy]	3.18	0.00–44.21	2.75	0.00–43.88	-13.52	0.009	0.213
V30 Gy [%]	68.55	14.54–100	62.22	0.5–100	-9.23	< 0.0001	0.384
V40 Gy [%]	52.99	0.00–100	47.09	0.00–100	-11.13	< 0.0001	0.321
**Axillary level II**							
Volume [ccm]	17.98	8.10–40.40	18.21	7.55–40.60	1.28	0.062	0.153
Dmean [Gy]	16.40	0.38–52.40	20.10	0.44–53.84	22.56	< 0.0001	0.453
D50% [Gy]	15.29	0.38–52.37	19.65	0.44–52.32	28.52	< 0.0001	0.423
Dmax [Gy]	34.54	0.58–57.94	38.80	0.69–60.11	12.33	< 0.0001	0.376
Dmin [Gy]	2.80	0.22–42.77	2.96	0.00–46.80	5.71	0.002	0.253
V30 Gy [%]	28.41	0.00–100	36.33	0.00–100	27.88	< 0.0001	0.415
V40 Gy [%]	17.74	0.00–100	22.94	0.00–100	29.31	< 0.0001	0.439
**Axillary level III**							
Volume [ccm]	8.61	4.00–19.40	8.28	3.80–20.10	-3.83	< 0.0001	0.445
Dmean [Gy]	3.06	0.21–40.48	3.84	0.25–39.23	25.49	< 0.0001	0.466
D50% [Gy]	2.38	0.21–47.19	3.23	0.25–44.97	35.71	< 0.0001	0.463
Dmax [Gy]	10.55	0.27–52.94	13.09	0.30–52.32	24.08	< 0.0001	0.492
Dmin [Gy]	0.80	0.13–3.58	0.87	0.16–3.24	8.75	< 0.0001	0.45
V30 Gy [%]	2.41	0.00–80.84	3.60	0.00–81.44	49.38	0.117	0.129
V40 Gy [%]	1.35	0.00–70.92	2.16	0.00–67.69	60	0.212	0.103
**Supraclavicular**							
Volume [ccm]	27.16	15.16–38.50	25.97	13.02–37.30	-4.38	< 0.0001	0.723
Dmean [Gy]	0.84	0.22–10.80	0.82	0.23–4.11	-2.38	0.002	0.26
D50% [Gy]	0.75	0.20–4.38	0.76	0.21–2.68	1.33	0.001	0.275
Dmax [Gy]	1.91	0.54–23.89	2.06	0.46–39.43	7.85	0.019	0.192
Dmin [Gy]	0.37	0–1.38	0.40	0–1.50	8.11	< 0.0001	0.375
V30 Gy [%]	0.00	0.00–0.00	0.00	0.00–0.00	0	1.000	0
V40 Gy [%]	0.00	0.00–0.00	0.00	0.00–0.00	0	1.000	0
**Internal mammary**							
Volume [ccm]	7.64	3.80–9.52	7.54	3.70–9.45	-1.31	< 0.0001	0.522
Dmean [Gy]	11.17	1.34–44.24	12.77	1.45–39.09	14.32	0.005	0.231
D50% [Gy]	9.90	1.25–49.93	11.97	1.47–47.57	20.91	0.001	0.273
Dmax [Gy]	35.21	1.74–64.58	36.02	1.99–59.48	2.3	0.144	0.12
Dmin [Gy]	1.84	0.55–3.65	1.87	0.60–3.89	1.63	0.127	0.126
V30 Gy [%]	11.03	0.00–86.55	14.28	0.00–78.74	29.47	0.019	0.193
V40 Gy [%]	4.83	0.00–82.66	6.45	0.00–67.61	33.54	0.020	0.192
**Breast right**							
Volume [ccm]	795.47	128.00–2762.68	801.19	121.80–2759.06	0.72	0.023	0.187
Dmean [Gy]	0.62	0.12–1.81	0.63	0.12–1.69	1.61	0.234	0.098
D50% [Gy]	0.53	0.00–1.020	0.54	0.00–1.70	1.89	0.661	0.036
Dmax [Gy]	5.34	0.38–45.77	5.12	0.55–40.31	-4.12	0.046	0.164
Dmin [Gy]	0.00	0.00–0.22	0.01	0.00–0.59	0	0.019	0.193
V30 Gy [%]	0.01	0.00–0.86	0.00	0.00–0.00	-1	0.317	0.082
V40 Gy [%]	0.00	0.00–0.28	0.00	0.00–0.00	0	0.317	0.082

Comparison of mean values (ranges) of DVH parameters for levels I-III, supraclavicular and internal mammary region and contralateral breast and relative changes in percent between DIBH and FB technique using two-sided Wilcoxon signed-rank test and effect size

*Abbreviation*: *DIBH* Deep inspiration breath hold, *DVH* Dose-volume histogram, *FB* Free breathing

**Fig. 2:**
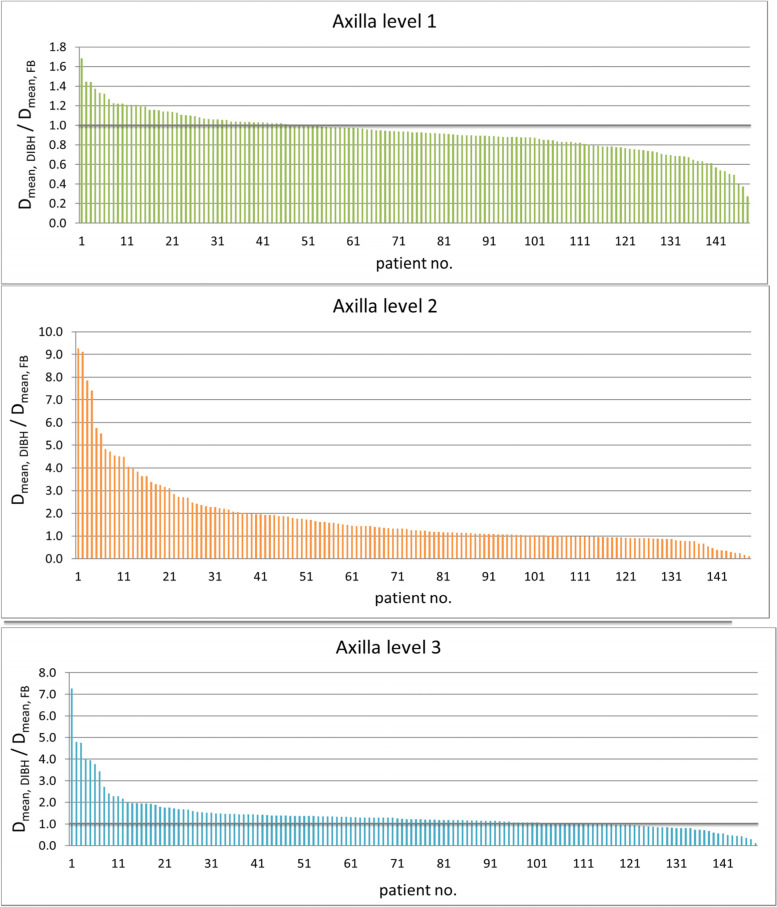
Ratio of D_mean_ for axillary level I-III in DIBH to FB position (Y-axis) in the entire cohort (X-axis, *n* = 148). Abbreviation: *DIBH*: deep inspiration breath hold; *FB*: free breathing

The mean dose (D_mean_) in level I, II and III in DIBH were 32.28 Gy (range 2.87–51.7), 20.10 Gy (range 0.44–53.84) and 3.84 Gy (range 0.25–39.23) vs. 34.93 Gy (range 10.52–50.40), 16.40 Gy (range 0.38–52.40) and 3.06 Gy (range 0.21–40.48) in the FB group (*p* < 0.0001 for all) (Fig. 2). In comparison to FB, D_mean_ for level I was reduced by 7.59% (effect size, *r* = 0.38) and increased for level II and III by 22.56% (*r* = 0.45), and 25.49% (*r* = 0.47).

The D50% in level I, II, and III in DIBH were 35.42 Gy (range 1.64–53.35), 19.65 Gy (range 0.44–52.32) and 3.23 Gy (range 0.25–44.97) vs. 39.02 Gy (range 2.75–52.05), 15.29 Gy (range 0.38–52.37) and 2.38 Gy (range 0.21–47.19) in the FB group (*p* < 0.0001 for all) (Table 2). Thus, in comparison to FB D50% for level I was reduced by 9.23% (*r* = 0.39), whereas it was increased for level II and III D50% by 28.52% (*r* = 0.42), and 35.71% (*r* = 0.46) (Table 2).

The mean values of V_30Gy_, and V_40Gy_ for level I in the DIBH cohort were decreased by 9.23% and 11.13% (*p* < 0.0001 for both, *r* = 0.38 and *r* = 0.32), respectively. There was an increase in the mean values of V30, and V40 for level II in DIBH 27.88% (*p* < 0.0001, *r* = 0.41) and 29.31% (*p* < 0.0001, *r* = 0.44) respectively. There was a non-significant increase in the mean values of V_30Gy_ and V_40Gy_ for level III with DIBH (Table 2).

The mean volumes of the lymph node levels were assessed in FB and DIBH. There were small, but significant volume decreases in DIBH for level III by 3.83% (*p* < 0.0001) but not for level II by 1.28% (*p* = 0.062).

### Supraclavicular and internal mammary region and contralateral breast

**Fig. 3:**
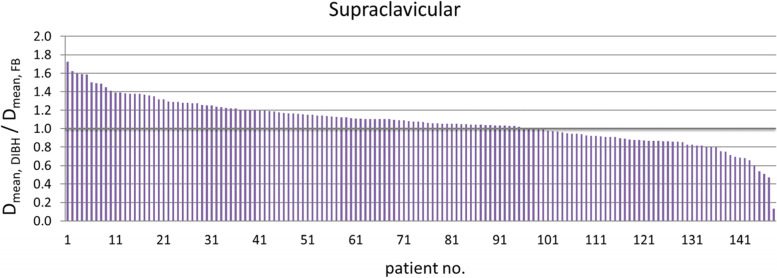
Ratio of D_mean_ for the supraclavicular region in DIBH to FB position (Y-axis) in the entire cohort (X-axis, *n* = 148). Abbreviation: *DIBH*: deep inspiration breath hold; *FB*: free breathing

The D_mean_ for the SC in DIBH was 0.82 Gy (range 0.23–4.11), as compared to 0.84 Gy (range 0.22–10.80) with FB (*p* = 0.002). This results in a D_mean_ reduction of 2.38% (*r* = 0.26) in DIBH (Fig. 3). The mean volumes of the SC in FB were 27.2 cm^3^ (range 15.2–38.5) vs. DIBH: 26 cm^3^ (range 13–37.3). This resulted with moderate volume decrease in DIBH for SC by 2.4% (*p* < 0.0001).

**Fig. 4:**
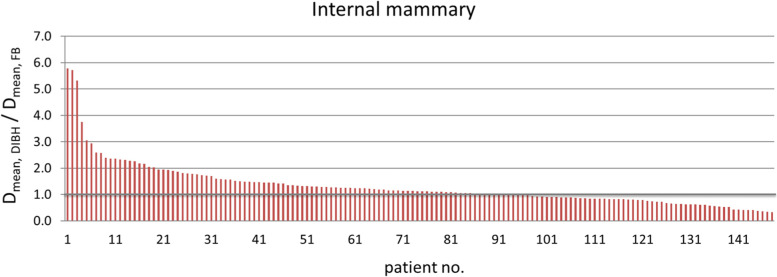
Ratio of D_mean_ for the internal mammary region in DIBH to FB position (Y-axis) in the entire cohort (X-axis, *n* = 148). Abbreviation: *DIBH*: deep inspiration breath hold; *FB*: free breathing

The D_mean_ for IM was 12.77 Gy (range 1.45–39.09) in DIBH vs. 11.17 Gy (range 1.34–44.24) in FB (*p* = 0.005). This yields a D_mean_ increase of 14.32% (*r* = 0.23) in DIBH (Fig. 4). The mean of V_30Gy_, and V_40Gy_ for IM in DIBH were by 29.47% (*p* = 0.019, *r* = 0.19) and 33.54% (*p* = 0.02, *r* = 0.19) (Table 2). The mean volume of the IM in FB was 7.6 cm^3^ (range 3.80–9.5) compared to 7.5 cm^3^ (range 3.7–9.5) in DIBH. This corresponded to a small, but significant decrease in volume in DIBH for IM by 1.3% (*p* < 0.0001).

Mean values for right (contralateral) breast were 795.5 cm^3^ (range 128–2762.7) for FB and 801.2 cm^3^ (range 121.80–2759.1) for DIBH. The mean relative difference between DIBH and FB was small (relative increase of 0.72%), but statistically significant (*p* = 0.023).

## Discussion

In this retrospective single-center analysis, we could demonstrate that the use of DIBH leads to significant changes in dose-volume parameters for patients with left-sided breast cancer treated with tangential 3DRT. For level I, DIBH lead to a decrease in incidental dose whereas DIBH was associated with increased doses for level II-III, supraclavicular and internal mammary lymph nodes.

Since publication of the ACOSOG Z0011-trial, there has been a controversial discussion regarding the radiation dose required for control of subclinical disease in the axilla [20]. Although the trial protocol mandated standard tangential irradiation, a subsequent retrospective analysis of 228 patients showed that more than half of the patients were treated with high tangents and 15% received supraclavicular irradiation [13]. A meta-analysis of prospective partial breast irradiation-trials demonstrated an increased risk of axillary recurrences with an odds ratio of 1.75 (95%-confidence interval 1.07–2.88), further suggesting that incidental axillary irradiation might contribute to locoregional control [21].

A recent systematic review identified 13 retrospective studies with a total of 475 patients which analyzed dosimetric parameters of axillary lymph node levels for patients planned for adjuvant whole breast radiotherapy [22]. There was considerable variation in axillary doses depending on the use of high tangents and radiation technique. For patients treated with standard tangential 3DRT, median dose in axillary levels I-III ranged from 22 to 43.5 Gy, 3 to 35.6 Gy, and 1 to 20.5 Gy, respectively [22]. In addition to these retrospective studies, Hildebrandt et al. recently published prospective data from the quality assurance program of the INSEMA-trial [23]. Incidental axillary doses were analyzed for 234 patients who underwent central plan review. Axillary level I and II were treated with a median of 72.8% and 39.9% of the prescribed dose in the breast, respectively. Level III only received a median dose of 16.4% of the breast dose. More than 25% of patients were treated with a median dose ≥ 95% of the breast dose in level I. Patients with a body mass index (BMI) ≥ 30 kg/m^2^ had significantly higher median doses in level I-III. Most patients received 3DRT (76.1%), while DIBH was only used in 1 patient [23].

DIBH is a standard technique for patients with left sided breast cancer due to a significant dose reduction of cardiac structures [24–26]. In 2019, the breast cancer expert panel of the German Society for Radiation Oncology (DEGRO) recommended the use of DIBH for the treatment of patients with left sided breast cancer [10]. However, DIBH is not only associated with a change in the position of the heart and lungs but also with changes in the chest wall and surrounding soft tissue [14, 15]. This may affect the incidental dose to axillary lymph node levels as well as the supraclavicular and internal mammary lymph nodes.

Borm et al. contoured axillary lymph node levels according to the RTOG atlas for 32 patients and compared incidental doses during DIBH and FB [15]. They demonstrated an overall three-dimensional movement of the axillary lymph node levels of 1.5 to 1.6 cm. The use of DIBH lead to a significant decrease in the incidental dose to level I and numerical, but mostly non-significant increases in the dose to level II and III [15].

Pazos et al. analyzed the influence of DIBH on doses to level I-III, supraclavicular and internal mammary nodes for 35 patients planned for radiotherapy of the breast or chest wall and the regional lymph nodes. Three-dimensional movement was between 0.79 cm for level I and 1.44 cm for internal mammary nodes. DIBH led to a significant decrease in the dose to level I and II compared to FB, however there were no significant differences for the other regions of interest [14].

In our analysis, the largest absolute changes were observed for level I with a decrease of 2.65 Gy (from 34.93 Gy to 32.28 Gy) and for level II with an increase of 3.7 Gy (from 16.40 Gy to 20.10 Gy). However, the clinical relevance may be more adequately addressed by comparing exposure to higher doses, such as V_40Gy_ and V_30Gy_. While only small amounts of level II were exposed to these doses, DIBH led to an absolute decrease in the V_40Gy_ and V_30Gy_ of 6.3% and 5.9% for Level I, respectively. Level III as well as the supraclavicular and the internal mammary nodes were only minimally exposed to incidental irradiation. The observed changes, though in some cases statistically significant, may not be clinically relevant due to relatively small absolute differences. Compared to the analysis by Borm et al. [15], the changes in D_mean_ and V_40Gy_ and V_30Gy_ are somewhat smaller in magnitude. Contrary to their findings, we saw an increase in all dosimetric parameters for level II as well as D_mean_ to level III, but nor for V_40Gy_ and V_30Gy_ for level III. We observed considerably lower doses to level II and III, for example for D_mean_ of level II: 16.40 Gy (FB) and 20.10 Gy (DIBH) in our analysis compared to 23.7 Gy (FB) and 24.1 Gy (DIBH) for Borm et al. [15]. This may be related to differences in treatment planning and DIBH-technique. While we used a surface scanning-approach with a pre-defined gating window of 5 mm, the real-time position management system (RPM, Varian Medical Systems, Palo Alto, CA) was used by Borm et al. [15]. No details regarding the gating window were provided.

Our analysis represents the largest cohort both in terms of dosimetric analysis in FB and in DIBH. We included intraindividual comparisons based on CT-scans in FB and DIBH for each patient. To exclude confounding by different radiotherapy techniques, only tangential 3DRT-plans were analyzed. It has been previously shown that IMRT or VMAT can also significantly impact incidental dose to the axilla [27–29]. Thus, our data cannot be extrapolated to patients receiving a combination of DIBH and IMRT/VMAT. Interindividual differences in anatomy may be responsible in part for dosimetric variability. Furthermore, compliance with breath hold as well as the quality of coaching for DIBH and the depth of inspiration may have an impact on anatomical changes and the position of lymph node areas in DIBH. Unfortunately, information on BMI was not available from the patient charts and could thus not be analyzed. The subgroup of patients with mastectomy was too small to provide reliable estimates for statistical comparisons. About 23% of patients in our analysis received hypofractionated radiotherapy, which in itself may lead to a decreased biologically effective dose to the unintentionally exposed axilla [30].

In conclusion, we could demonstrate a significant variability in incidental dose exposure to regional lymph node areas in patients with left-sided breast cancer by the use of DIBH. Further studies are needed to determine the clinical significance of these findings and to establish predictors of dosimetric changes based on patient-related factors.

## Data Availability

The data used in this analysis are available with the authors’ permission.

## Abbreviations

AL: Axillary lymph nodes
ALND: Axillary lymph node dissection
BCS: Breast conservation surgery
CI: Confidence interval
3DRT: 3D-conformal radiotherapy
DVH: Dose-volume histogram
DIBH: Deep inspiration breath hold
ER: Estrogen receptor
FB: Free breathing
HER2: Human epidermal growth factor receptor 2
IM: Internal mammary lymph nodes
IMRT: Intensity-modulated radiotherapy
IORT: Intraoperative radiotherapy
MV: Megavolt
OS: Overall survival
PR: Progesterone receptor
RT: Radiotherapy
SC: Supraclavicular lymph nodes
SGRT: Surface image guided radiotherapy
SIB: Simultaneous integrated boost
SLND: Sentinel lymph node dissection
TNBC: Triple-negative breast cancer
VMAT: Volumetric modulated arc therapy
WBI: Whole breast irradiation
